# Application of Medicinal Mushrooms for the Treatment of Peripheral Nerve Injury: A Systematic Review

**DOI:** 10.3390/medsci14010042

**Published:** 2026-01-16

**Authors:** Nurul Aini Binti Taib, Zolkapli Bin Eshak, Hussin Bin Muhammad, Muhammad Danial Bin Che Ramli

**Affiliations:** 1 School of Graduate Studies, Management and Science University (MSU), Shah Alam 40010, Selangor, Malaysia; nurrulaini@outlook.com; 2 Faculty of Pharmacy, Universiti Teknologi Mara (UITM), Puncak Alam 40100, Selangor, Malaysia; zolkapli_eshak@uitm.edu.my; 3 Toxicology & Pharmacology Unit, Herbal Medicine Research Centre, Institute for Medical Research, National Institutes of Health, Setia Alam, Shah Alam 40170, Selangor, Malaysia; hussin.m@moh.gov.my; 4 Faculty of Health and Life Sciences, Management and Science University, Shah Alam 40010, Selangor, Malaysia

**Keywords:** mushrooms, peripheral nerve injury, functional recovery, neuroprotection, sciatic nerve

## Abstract

**Background/Objective**: Current treatments for peripheral nerve injury (PNI) lack robust evidence to suggest complete recovery; hence, alternative therapeutics offer new opportunities to develop more effective protocols. Mushroom species and their related components are considered potential candidates for peripheral nerve repair, but their specific effects and underlying mechanisms are not fully understood. This systematic review presents the available evidence on the use of mushroom species for PNI therapy, including the bioactive components and mechanisms of action. **Methodology**: A comprehensive literature search in three databases (PubMed, Scopus, and Google Scholar) led to the synthesis of 11 records published between 2010 and 2024. Qualitative analysis revealed the neuroregenerative potential of four mushrooms: *Amanita muscaria* (*n* = 2), *Hericium erinaceus* (*n* = 5), *Lignosus rhinocerotis* (*n* = 3), and *Flammulina velutipes* (*n* = 1), with aqueous extracts as the most common type of ingredients used (*n* = 4) relative to specific components such as muscimol, polysaccharide, Erinacine S, and nerve-guided conduits (NGCs). **Results**: These mushroom-derived treatments enhanced the migration of Schwann cells mainly via the FGF-2 signalling and MAPK pathway. In vivo studies also revealed the ability of *H. erinaceus*, *A. muscaria*, and *L. rhinocerotis* to promote peripheral nerve repair and functional recovery, with evidence suggesting the role of neurotrophic factors, anti-apoptotic signalling, and pro-inflammatory substances. *H. erinaceus* was identified as the most promising for potential clinical applications, given the stronger evidence-based data and its relatively safer components compared to *A. muscuria* and other mushroom species. **Conclusions**: Despite presenting the potential use of mushrooms in managing PNIs, the existing approaches need to be subjected to clinical research to accelerate the development of future therapeutics and preventive measures for PNIs.

## 1. Introduction

Peripheral nerve injury (PNI) induces restricted activity and lifelong disability in affected animals and humans [1]. PNI can also lead to partial or complete loss of sensory, motor, and autonomic functions at denervated sites, resulting in temporary or permanent disability [1,2]. Vehicular and traumatic accidents, lacerations, and iatrogenic affections constitute the majority of PNIs, which often reduce the quality of life in affected persons [3,4,5]. In addition, individuals affected with PNI experience serious functional deficits with significant economic impacts [2]. Given the public health and economic implications, there is a growing interest in developing effective treatment and management modalities for PNIs and neurogenerative disorders.

Remarkable improvement has been observed in microsurgical treatment for nerve injuries over the past decades; however, the outcome of PNI repair remains unsatisfactory [6]. Animal models such as rabbits, rats, mice, dogs, cats, sheep, and pigs have been utilised for assessing peripheral nerve regeneration and repair before implementing the findings in clinical practice [7]. Available evidence from prior research reflects the scarcity of effective approaches for nerve repair and regeneration and ensuring complete functional recovery [8]. While nerve autograft is considered the gold standard for ameliorating defects of the peripheral nerve, the technique is limited based on the low availability of donor nerves, donor site morbidity, and unsatisfactory recovery rates [9]. As a result, alternative approaches such as medicinal plants have been proposed to enhance nerve repairs, serving as key components in complementary medicine [10]. PNI usually causes neuropathic pain, with patients resorting to complementary medicines such as nutritional supplements and herbal products when conventional therapies are ineffective [11]. Hence, novel plant-based treatments and combinatorial modalities are being explored to elucidate their capacities in enhancing axonal regeneration and functional recovery [10,11].

Mushrooms are well-documented as optimal sources of nutrients and health-enhancing components [12,13,14]. More than 2000 species of medicinal and edible mushrooms are available worldwide [15]. Several studies have also identified the medicinal properties of various mushrooms, especially those belonging to the Phylum Basidiomycota [15,16]. This group of mushrooms contain a diverse number of bioactive compounds, making them highly valuable and crucial components of pharmaceutical products [16]. Among the drivers for the growing research on mushrooms are their neuroprotective and antioxidant properties [16]. Examples of well-known species of mushrooms in translational medicine research, particularly studies on neurodegenerative diseases, include Sarcodon, Cyathus, Hericium, Antrodia, Ganoderma, and Pleurotus [15,16].

Despite the increasing research and use of mushroom-derived treatments for peripheral nerve repair and regeneration, the specific effective constituent and their potential source for promising drug candidates are not well understood. Moreover, the results from available studies, especially in terms of neurogenerative effects, functional recovery, and their comparative effectiveness, are yet to be systematically analysed. A comparative analysis of mushroom-derived treatments is pertinent to identify the potent candidates for ameliorating neurogenerative defects, as well as understanding their mechanisms of action and potential application in clinical settings.

A number of reviews on treatment modalities for PNI have been published in the last decade. For example, Choo et al. [17] focused on the generality of PNI research and the mechanisms to optimise peripheral nerve regeneration, whereas Yadav et al. [12] explored the bioactive potentials of mushrooms and the underlying mechanisms for their neuroprotective properties. Yow et al. [10] reviewed complementary and alternative medicines and potential use in peripheral nerve regeneration. The common limitation in these reviews is either the lack of a systematic approach or the failure to focus specifically on mushroom species as a therapeutic modality for PNI. A systematic review of previous research offers the opportunity to compare the results and identify the research gaps to be bridged in future investigations. Hence, the present study aims to analyse the available evidence and the mechanisms of action regarding mushrooms and their bioactive potentials for PNI therapy.

## 2. Materials and Methods

We used the modified version of the PRISMA guidelines for this review, which entails synthesising the results from relevant studies without performing statistical analysis [18]. This modification was performed to align the procedures with the broad review objectives and the expected variation in the outcomes explored in the relevant studies. Thus, the main elements omitted from the PRISMA guidelines were those required for meta-analysis. All other items in the guidelines were strictly followed.

The search databases were PubMed, Scopus, and Google Scholar, and each database was searched using combinations of the three groups of keywords, aligning with the review objectives and main research components. The specific keywords under each group are provided in Table 1. Table 2 presents the search strings used for the literature search in each database.

The databases were accessed between July 2024 and August 2024. A further manual search was performed based on the references of the studies identified from the primary search. The primary objective of the search was to retrieve any article that investigated the therapeutic effect (i.e., neurodegenerative, neuroprotective, and/or functional recovery) of mushroom species and their ingredients in PNI, either in vitro or in vivo. In addition, studies reporting the underlying mechanisms were identified from the electronic and manual searches. This SLR was not registered in PROSPERO or any other research protocol registration platforms.

### 2.1. Study Selection

Two independent reviewers screened all titles, abstracts, and full-text articles. The reviewers were trained in systematic literature search and data extraction from research articles. Thus, interobserver assessment was not performed to determine the agreement level and the possibility of selection bias. In cases of non-consensus and disagreement between the two reviewers, a third independent review was obtained for resolution.

All the retrieved articles were subjected to the following eligibility criteria: original research articles published between 2010 and 2024, written in English, and focusing on the use of mushroom species, products, or extracts for the treatment of peripheral nerve injuries. Furthermore, interventional in vitro or in vivo studies involving the use of single or multifaceted approaches, including at least one medicinal mushroom, were included in the review. Only randomised controlled trials (RCTs) and quasi-experimental research articles were considered since we aim to elucidate the effects of various treatment modalities for PNI. Animals or humans fulfilling the criteria of exposure to any of the treatment modalities described earlier for the management of PNI were included in the review. Articles not related to PNI, not involving mushroom species or related products, meta-analyses, review articles, abstracts, editorial/letters, case reports, and conference proceedings were excluded.

### 2.2. Data Extraction and Data Analysis

One of the reviewers extracted the relevant data from each article by using predefined selection criteria, which was subsequently cross-checked by a second reviewer. The items extracted comprised information related to the specific mushroom species, components or ingredients used for the treatment, dose, study design and type of experiment, method of administration, experimental subjects, assessment of functional and sensory recovery, and mechanisms of action explored. Since the reviewers are conversant with the research topic and well-trained in systematic literature search, inter-rater agreement was not computed during the data extraction process.

In terms of data analysis, only descriptive analyses were performed in this review. Meta-analysis and pooled estimates were not computed due to methodological heterogeneity and the diverse substances explored in the reviewed studies. Moreover, the investigated outcomes differed between most of the reviewed studies, thus violating the underlying condition required for robust meta-analyses.

### 2.3. Assessment of Methodological Quality and Risk of Bias

The quality of each article was evaluated independently by two reviewers using a 10-domain risk of bias assessment tool, Systematic Review Centre for Laboratory Animal Experimentation (SYRCLE) [19]. The tool was selected following a discussion among the authors regarding the most appropriate questions to be asked when assessing the quality of pre-clinical studies. Overall, the tool investigates the adequate generation, blinding, and application of the allocation sequence or randomisation, blinding of the research procedures (i.e., drug administration and outcome assessors), the avoidance of selective outcome reporting, random housing, and methods used for outcome assessments. A third reviewer was invited to resolve cases of disagreement between the quality assessors.

## 3. Results

### 3.1. Descriptive Findings

**Figure 1:**
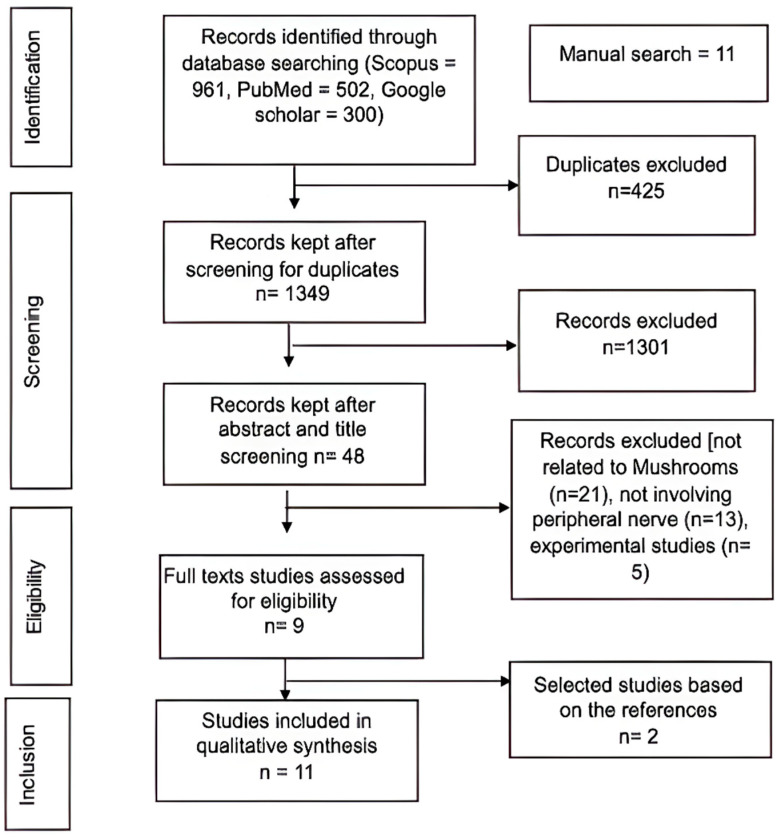
PRISMA flow chart on literature selection based on inclusion/exclusion criteria to identify studies investigating the use of mushroom species for PNI treatment.

The literature search results across the three search engines are summarised in Figure 1. Combined with 11 other records synthesised from a manual search, the remaining records were screened according to the eligibility criteria. This led to the further removal of 324 articles, leaving 11 records for the final analysis. As shown in Table 3, the studies explored the neuroregenerative potential of four various mushrooms: *Amanita muscaria* (*n* = 2), *Hericium erinaceus* (*n* = 5), *Lignosus rhinocerotis* (*n* = 3), and *Flammulina velutipes* (*n* = 1).

Seven of the reviewed studies attempted to either isolate or characterise the specific bioactive compounds responsible for the neuroprotective and ameliorative effects, ranging from polysaccharides [9,20], to muscimol [20], Erinacine S [21], *N*-de phenylethyl isohericerin (NDPIH) and its hydrophobic derivative, hericene A [15], and nerve-guided conduits (NGCs) [22]. Two of the seven studies investigated a combination of two bioactive compounds [15,23]. The cell lines used were DRG neuron culture [21,24], cortical neuron cultures [21], pheochromocytoma (PC-12) Adh cell lines [23], and cultured hippocampal neurons [15]. The remaining studies involved either parts or whole mushroom extracts [14,24,25,26,27].

The two studies on *A. muscaria* were in vivo, conducted in SD rats, and the mechanisms of action were explored [9,20]. Meanwhile, research on *H. erinaceus entailed* three in vitro and two in vivo studies, and only three reported the mechanisms of action. As for *L. rhinocerotis*, two studies used an in vitro model [14,23], whereas Farha et al. [27] performed an in vivo experiment in SD rats. None of the studies investigated the underlying mechanisms. The single study on *F. velutipes* (FVC) used a mixed method, comprising in vitro and in vivo experiment models [21]. The researcher also proceeded by investigating the potential role of the growth-associated protein 43 (GAP-43) in the neuroregenerative properties of the nerve-guided conduits (NGCs) (labelled FVC).

**Table 3:** Descriptive characteristics of the studies included in this review.

Type of Mushroom	Ingredients	Experimental Model	Effective Concentration and Method of Administration	Type of PNI	Biological Effects	Mechanism of Action	Reference
*Amanita muscaria*	Muscimol	In vivo (SD rats)	Direct injection of 400 μg/mL of muscimol into a hole drilled in the right L5 DRG	Sciatic crush injury for the experimental group and nerve mobilisation of the sciatic nerve without crush injury in the control group	Promoted peripheral nerve regeneration in rats with SNI (prevented the development of thermal and mechanical hypersensitivity and mechanical allodynia, improved basal membrane integrity, and increased).	Normalisation of PMP22 protein expression level by GABAergic modulation in the ipsilateral DRG	Naik et al. [20]
	Aqueous extract	In vivo (SD rats)	Daily oral administration at 10 mL per kg	Sciatic nerve injury	Promoted peripheral nerve regeneration in rats following peroneal nerve crush.	Protein synthesis and activation of several signalling pathways, such as Akt, MAPK, c-Jun, and C-fos	Wang et al. [9]
*Hericium erinaceus*	Polysaccharide	In vivo (SD rats)	Daily oral administration of polysaccharide from *H. erinaceus* at 30 mg/mL/kg body weight/day) for 14 days	Peroneal crush injury	Promoted sensory functional recovery following peroneal nerve crush in rats (reduced withdrawal reflex latency).	Activation of Akt and p38 MAPK signalling and increasing expression of RECA-1	Wong et al. [25]
	Aqueous extract	In vivo (SD rats)	Daily oral administration of aqueous extract of fresh fruit bodies at 30 mL/kg for 14 days	Peroneal nerve crush	Promoted peripheral nerve regeneration post-peroneal nerve crush (increased PFL, improved axon morphology and development of the neuromuscular junction).	Activation of Akt and p38 MAPK signalling and increasing expression of RECA-1. Restoration of the integrity of the blood–brain barrier	Wong et al. [26]
	Erinacine S	In vitro (rats and mice cortical neuron cultures)	1 ug/mL, 100 ng/mL, 10 ng/mL, and 1 ng/mL were inoculated into the primary neurone and DRG cultures	DRG neuron cultures	Erinacine S depicted significant effects on neurite outgrowth in neurons of the peripheral nervous system. Cortical neuron attachment on CSPGs, length of neurites, and axon regeneration were also increased.	The accumulation of neurosteroids, pregnenolone and progesterone, was induced by Erinacine S, which is responsible for neurite outgrowth enhancement	Lin et al. [21]
	Hot aqueous extracts	In vitro (primary cultures of DRG neurons from mice)	Treatment groups comprised 25 μg/mL of HE, 50 ng/mL of NGF, and a combination of both HE and NGF. Pre-treatment with PBS was performed before axon transection	DRG neuron cultures	Both NGF and HE depicted neuroprotective and neurodegenerative effects on axotomized peripheral sensory neurons; the effects were maximised when both treatments were combined.	NA	Üstün & Ayhan [24]
	*N*-de phenylethyl isohericerin (NDPIH), and its hydrophobic derivative, hericene A	In vitro (cultured hippocampal neurons from mice)	Direct inoculation of extracts into cultured hippocampal neurons	Hippocampal neuron cultures	The combination of *N*-de phenylethyl isohericerin (NDPIH) and its hydrophobic derivative, hericene A, was highly effective in promoting marked axon outgrowth and neurite branching in the cultured cells, reflecting potent neurotrophic activity. NDPIH-induced neurotrophic activity was partly prevented by ANA-12’s inhibition of tropomyosin receptor kinase B (TrkB), indicating a potential relationship with BDNF signalling.	ERK1/2 signalling was activated by NDPIH without the presence of TrkB in HEK-293T cells—an event that was insensitive to ANA-12 in the presence of TrkB	Martínez-Mármol et al. [13]
***Lignosus rhinocerotis***	Aqueous extract	In vivo (SD rats)	Daily oral administration of aqueous extract at 500–1000 mg/kg	Complete sciatic crush injury	Promoted motor and sensory functional recovery in rats with SNI (improved WRL and toe-spreading reflex).	NA	Farha et al. [27]
	Sclerotium extract combined with nerve growth factor (NGF)	In vitro (PC-12Adh cell line)	Inoculation of 20 ug/mL of sclerotium extract and 30 ng/mL of NGF into PC-12 ADH cell lines	PC-12 ADH cell lines	Treatment with aqueous extract alone induced neurite outgrowths of 24.4% at 20 μg/mL (*w*/*v*), whereas when combined with 30 ng/mL (*w*/*v*) of NGF, the neurite outgrowth increased to 42.1%.	NA	Lee-Fang Eik et al. [23]
	Hot aqueous and ethanolic extracts, and crude polysaccharides	In vitro (rat pheochromocytoma (PC-12) cells)		Rat pheochromocytoma (PC-12) cells	The hot aqueous extract exhibited neuritogenic activity comparable to NGF in PC-12 cells. However, the extracts and crude polysaccharides stimulated neuritogenesis without stimulating the production of NGF in PC-12 cells. No significant difference in protein expression in NGF- and hot aqueous extract-treated cells for both total and phosphorylated p44/42 MAPK.	The extracts stimulated neurogenic activity in PC-12 cells that mimicked NGF activity via the MEK/ERK1/2 signalling pathway	Seow et al. [14]
*Flammulina velutipes*	Nerve-guided conduits (NGCs) (labelled FVC)	In vitro and in vivo (SD rats)	Direct inoculation into cell lines and oral administration to rats	Sciatic nerve defects	FVC effectively stimulated nerve functional recovery and axonal outgrowth when applied to ameliorate critical-sized sciatic nerve defects in a rat model.	Growth-associated protein 43 (GAP-43) was increasingly expressed in the FVC group compared to the autograft group. FVC upregulated the phosphorylation of signal transducer and activation of transcription-3 (P-STAT3), thereby leading to the secretion of GAP-43	Chen et al. [22]

### 3.2. Quality Appraisal Results

Table 4 presents the methodological quality of the studies. Eight of the studies demonstrated a high risk of bias (i.e., low quality), mainly for not fulfilling the various domains: adequate allocation sequence, allocation concealment, random housing of animals, and blinding of the research conductors and outcome assessors. However, most studies ensured that baseline groups were similar and performed adequate adjustments for missing data and were free of selective outcome reporting. Only three studies demonstrated a moderate risk of bias [21,25,26], indicating that no study recorded a low risk of bias.

### 3.3. Peripheral Nerve Regenerative Properties of Amanita muscaria

One study involved the use of a specific ingredient, muscimol [20], while the other study used an aqueous extract of the mushroom, *Amanita muscaria* [25]. Both studies were in vivo, characterised by the oral administration of aqueous extract [25] and direct application of muscimol to the right L5 DRG of SD rats [20]. Muscimol facilitated peripheral nerve regeneration in rats with sciatic nerve injury by preventing the development of mechanical and thermal hypersensitivity, as well as mechanical allodynia. The ingredient enhanced basal membrane integrity [20]. The underlying mechanism entailed the normalisation of PMP22 protein expression level via GABAergic modulation in the ipsilateral DRG. On the other hand, aqueous extract from *Amanita muscaria* promoted peripheral nerve regeneration post-peroneal nerve crush through protein synthesis and activating diverse signalling pathways such as Akt, MAPK, c-Jun, and C-fos [25].

### 3.4. Peripheral Nerve Regenerative Properties of Hericium erinaceus

Among the four studies in this category, two each involved the use of aqueous extract [24,27] and specific ingredients: polysaccharide [26] and Erinacine S [21]. The oral administration of 10–20 mL/kg to SD rats resulted in an increased level of peripheral nerve regeneration following peroneal nerve crush, characterised by improved axon morphology, increased PFL, and neuromuscular junctional development [26]. Ustun and Ayhan [24] compared the neuroregenerative effects of hot aqueous extracts and NGF on axotomized peripheral sensory neurons cultured from mice. Although 25 ug/mL of *Hericium erinaceus* extracts exhibited significantly higher neuroprotective activity relative to the 50 ng/mL of NGF, the effects were maximised in the group treated with a combination of HE and NGF at 25 ug/mL and 50 ng/mL, respectively.

Oral administration of HE polysaccharide to SD rats at 30 mg/mL/kg led to enhanced sensory functional recovery post-peroneal nerve crush, evidenced by reduced withdrawal reflex latency [26]. The underlying mechanisms identified were the activation of p38 MAPK and Akt signalling and increased expression levels of RECA-1. In vitro study involving direct inoculation of 100 μg/mL to 10 ng/mL of Erinacine S to DRG cultures revealed a significant increase in cortical neurone attachment, length of neurites, and axonal regeneration in injured neurons [21]. Neurite outgrowth in this study was linked to the accumulation of neurosteroids.

**Figure 2:**
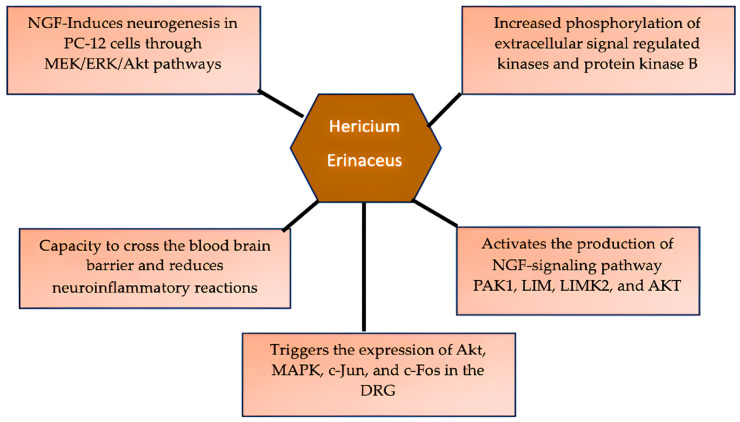
Mechanistic pathways for the neuroprotective effects of *Hericium erinaceus* based on the findings from the reviewed studies. Notes: MAPK = mitogen-activated protein kinase; ERK = extracellular regulating kinase; PAK1 = p-activated kinase.

Based on the ability of *H. erinaceus* to promote neurite outgrowth in hippocampal neurons, Martínez-Mármol et al. [13] identified biologically new active compounds in the mushroom known as *N*-de phenylethyl isohericerin (NDPIH) and its hydrophobic derivative hericene A. The bioactive compounds were highly effective in promoting marked axon outgrowth and neurite branching in the cultured cells, reflecting potent neurotrophic activity. NDPIH-induced neurotrophic activity was partly prevented by ANA-12’s inhibition of tropomyosin receptor kinase B (TrkB), indicating a potential relationship with BDNF signalling. Their results revealed that a complementary neurotrophic pathway independent of TrKB with converging downstream ERK1/2 activation constituted the mechanism of action for NDPIH. In the in vivo study, oral administration of crude extract and hericene A to mice led to a marked increase in neurotrophin expression and downstream signalling, leading to enhanced hippocampal memory. The researchers concluded that hericene A acts via a novel pan-neurotrophic signalling pathway, resulting in enhanced cognitive performance. Figure 2 depicts the identified mechanistic pathways of *H. erinaceus* as a potential neuroprotective agent.

### 3.5. Peripheral Nerve Regenerative Properties of Lignosus rhinocerotis

Three studies in this category involved oral administration of aqueous extract to SD rats [28], a combination of sclerotium extract and NGF in PC-12 Adh cell lines [23], and a combination of hot aqueous and ethanolic extracts and crude polysaccharides in PC-12 Adh cell lines [14].

Farha et al. [27] found that aqueous extract from *L. rhinocerotis* promoted sensory and motor function recovery in rats with artificially induced SNI, evidenced by improved WRL and toe-spreading reflex. Likewise, the combined effects of 20 ug/mL of *L. rhinocerotis* extract and 30 ng/mL of NGF induced significantly greater neurite outgrowths (42.1%) compared to aqueous extract alone (24.4%) [23].

Seow et al. [14] found that neuritogenesis was markedly stimulated in PC-12 cells upon inoculating hot aqueous and ethanolic extracts, and crude polysaccharides of *L. rhinocerotis* sclerotium. None of the concentrations of the extracts and crude polysaccharides were cytotoxic to PC-12 cells. Comparable neuritogenic activities were stimulated by the hot aqueous extract (25 μg/mL) and NGF (50 ng/mL). It was concluded that the neuritogenic activity may be mediated via the phosphorylation of TrkA receptors and the ERK1/2 signalling pathway in PC-12 cells.

### 3.6. Peripheral Nerve Regenerative Properties of Flammulina velutipes

Only one study explored the neuroprotective and regenerative effects of *Flammulina velutipes* for PNI and sciatic nerve defects [22]. In the in vitro study, F. *velutipes* conduits (FVC) were constructed and inoculated into cell lines from SD rats and observed for neuroregenerative activities. An in vivo study was also conducted in a rat model via oral administration and observed for the repair of nerve defects. FVC effectively stimulated axonal outgrowth and nerve functional recovery. Evidence of increased expression levels of growth-associated protein 43 (GAP-43) was exhibited in the treatment group, but the effect was not superior to the autograft group.

## 4. Discussion

Based on the 11 studies included in this review, we identified four species of mushrooms that have been explored in both in vivo and in vitro studies to elucidate their effects on PNIs. The mushroom explored in most of the studies was *Hericium erinaceus*, especially the aqueous extract 24 and specific ingredients, namely, polysaccharide and Erinacine S [21]. The extensive investigation of *H. erinaceus* for its neuroprotective and ameliorating effects on PNIs might stem from its unique bioactive compounds, erinacines and hericenones, which stimulate NGF synthesis, thus facilitating neuronal growth, repair, and regeneration [3]. The Lion’s mane is also the most widely reported among all culinary mushrooms, given its therapeutic activities related to the enhancement of brain and nerve health [3].

In the reviewed studies, promising findings were gleaned from the oral administration of 10–20 mL/kg of aqueous extracts to SD rats with peroneal nerve crush, evidenced by restoration of axon morphology, enhanced PFL and neuromuscular development. Furthermore, the neuroprotective activity of aqueous extract from *H. erinaceus* was maximised when combined with 50 ng/mL of NGF [24]. These promising results, characterised by neuronal survival and neurite outgrowth in the injured neuron model in vitro, are consistent with prior research [28,29,30]. For instance, HE extracts stimulated NGF synthesis and promoted NGF-induced neurite outgrowth in diverse cell types [31,32]. HE also reduces oxidative stress and modulates anti-inflammatory reactions [33]. Therefore, this review reflects that HE is a potent candidate for treating PNI, given its protective activity on neuronal death and improving nerve function.

The oral administration of HE polysaccharide to SD rats was equally accompanied by enhanced sensory functional recovery [26], which was linked to the activation of p38 MAPK, Akt signalling, and increased expression levels of RECA-1. The activation of these pathways persisted for one week post-injury until the onset of axonal regeneration. These mechanistic pathways are consistent with prior studies, whereby HE promoted neuronal survival, facilitated nerve regeneration and neurite outgrowth via the TrKA/Erk1/2 pathway [20,28]. The expression of Akt in DRG and nerves appears to be necessary to maintain cell survival, and an upregulation is critical to prevent mass cell death that may result from nerve injury [28]. Namikawa et al. [29] also observed a transient increase in ERK1 mRNA in motor neurons during hypoglossal nerve regeneration in rats, strengthening the evidence that MAPK is pertinent for neurite outgrowth even in vivo.

Polysaccharides are responsible for several functions in various cell types, such as muscle reinnervation, peripheral nerve regeneration and neuritogenesis in response to a sciatic nerve injury [30,31,32]. Polysaccharides derived from *H. erinaceus* can attenuate thermal hyperalgesia induced by crush nerve injury and promote reflex responses due to the reinnervation of sensory receptors by their primary axons. They also effectively restore the integrity of the blood–brain barrier (BNB) post-crush injury [30]—reflecting a key reaction in nerve regeneration. Nevertheless, the reviewed studies are yet to identify and characterise the polysaccharides and other related compounds which may initiate signalling cascades following axonal injury, and modify strategies to improve regeneration.

Another possible pathway for the neuroprotective effect of Erinacine S, an ingredient from *H. erinaceum*, is the accumulation of neurosteroids, leading to increased cortical neurone attachment, axonal regeneration, and neurite outgrowth in injured neurons [21]. The development of neurodegenerative diseases is generally associated with a decline in neurosteroids. Hence, the increased synthesis of these specific steroids promotes neurite outgrowth, myelination, and neuronal survival, thereby protecting neurons against apoptosis and inducing neurogenesis [33,34].

The next mushroom species identified in this review was *A. muscaria*, with one study each reporting the use of its ingredient, muscimol, and aqueous extract in vivo [20,25]. Muscimol prevented the onset of mechanical and thermal hypersensitivity, thereby facilitating peripheral nerve regeneration. Similar results were observed following the administration of aqueous extract from *A. muscaria* to the right L5 DRG of SD rats with induced sciatic crush injury [25]. The underlying events comprised maintenance of basal membrane integrity, normalisation of PMP22 protein expression level [25], protein synthesis, and activation of several signalling pathways such as Akt, MAPK, c-Jun, and C-fos [26]. These results align with several prior studies conducted on other mushroom species, such as *Ganoderma lucidum*, *Hirsutella sinensis*, and *Antrodia cinnamomea*, characterised by the activation of intracellular signalling kinases ERK, p38, and JNK, which are related to peripheral nerve regeneration [35].

Muscimol is a potent gamma-aminobutyric acid (GABA receptor agonist, which explains its modulating effect on the ipsilateral DRG and restoration of PMP22 protein expression in the sciatic nerve. These events improve basal membrane integrity in nerve fibres and catalyse nerve fibre regeneration. Thus, the robust effect of muscimol entails a combination of its higher binding affinity to alter GABAA receptors in DRG neurons, as their activation induces presynaptic inhibition, particularly at the dorsal spinal horn [35]. Other mechanisms suggested in the literature for the neuroprotective effects of muscimol include ROS scavenging; influence of the metabolism at the mitochondrial level; GSH preservation; reducing glial fibrillary acidic protein (GFAP) expressions, which is pivotal in Parkinson’s disease (PD) [36,37].

The reported ameliorative effects of muscimol on PNI are promising for translational studies, given the availability of various GABAergic agents for human use and the simplicity of direct DRG application. However, it is most effective when utilised at the early stages of nerve injury [25]. In addition, A. muscaria contains the compounds muscimol and its biosynthetic precursor, ibotenic acid. Both compounds are highly toxic, with effects ranging from dizziness to dysphoria, visual hallucinations, ataxia, muscle fasciculation, seizures and coma, following ingestion of *A. muscaria* mushrooms [12,14]. A case of human death was reported recently in a 44-year-old man who died upon ingesting four dried caps of *A. muscaria* [38,39].

*L. rhinocerotis* is the next in line among the identified mushroom species as potential candidates for the treatment of PNI. Two studies involving the oral administration of aqueous extract from *L. rhinocerotis* either as a single treatment [27] or combined with NGF in PC-12 Adh cell lines [22] led to enhanced nerve function and greater neurite outgrowths compared to the controls. The latter study corroborates the findings by Ustun and Ayhan [24], whereby the neuroprotective effect of *H. erinaceus* was greater when combined with 50 ng/mL of NGF. Although these studies did not investigate the underlying mechanisms, *L. rhinocerotis* possesses a broad spectrum of immunomodulatory and neuritogenic activities linked to the presence of polysaccharides and their related protein complexes, glucans, peptides, proteases, alkaloids, and diterpenes [14,36,37]. Moreover, polysaccharides and β-glucan from *H. erinaceus* aqueous extract were found to play a neuroregenerative role in the peripheral nervous system [25]. These events have also been linked to the ability of *L. rhinocerotis* to relieve neuropathic pain and multifaceted effects on PNIs.

Despite the positive findings, the issue of low bioavailability in *L. rhinocerotis* necessitates the use of high dosages to attain therapeutic effects in vivo, thereby increasing the risk of side effects [40]. While the high dosage of aqueous extract (i.e., 1000 mg/kg) did not cause any toxic signs or mortality in the experimental animals, no improvement in motor function was observed. Further studies are required to determine the optimum dosage of *L. rhinocerotis* to achieve its maximal potential in peripheral nerve regeneration, as well as the active compounds involved in the stimulation of neurite outgrowth and functional recovery. One study in this review revealed the use of FVC, a nerve conduit developed from the mushroom, *Flammulina velutipes*, for its ability to promote peripheral nerve reinnervation. However, the efficiency of the device as a bridge to ameliorate critical-sized nerve defects was inferior compared to autologous nerve grafts. Despite the findings, the research presents new insight into the use of natural conduits in nerve tissue engineering [22].

Important limitations in the reviewed studies need to be considered when interpreting the findings, particularly in terms of study designs, risk of bias, lack of standardisation of mushroom extracts, and predominant use of animal data. Most studies obtained low-quality ratings, with the predominant issues relating to the experimental methods. Apart from using a small number of samples, the majority of studies did not explain the sample size estimation and methods employed to reduce bias in terms of sequence allocation, administration of intervention, and outcome assessment. These methodological limitations affect the study’s reproducibility and the reliability of the findings.

The lack of standardisation of mushroom extracts is also a notable limitation, as several studies focused on the use of whole mushroom extracts rather than isolating and characterising specific bioactive compounds responsible for the therapeutic effects. This approach limits the understanding of the precise mechanisms underlying peripheral nerve repair. Include future research to prioritise the identification and profiling of individual bioactive metabolites, such as polysaccharides, terpenoids, or phenolic compounds, using advanced analytical techniques like HPLC, LC-MS/MS, or NMR spectroscopy. Such compound-level investigations could help pinpoint the active agents, enable dose standardisation, and facilitate the development of targeted therapeutics with higher efficacy and reproducibility.

## 5. Conclusions and Future Recommendations

PNI remains a significant health problem, and the effectiveness of present treatment modalities is highly subjective. Therefore, in order to discover novel and implement appropriate methods of managing this neurodegenerative disorder, substantial effort is required. Current studies have depicted promising findings in using various treatments derived from mushroom species for treating PNI, and their potential may be improved when the treatments are combined, particularly with NGF. Accumulated evidence from in vivo and in vitro studies has delineated the neuroregenerative properties of three mushroom species, namely *H. erinaceus*, *A. muscaria*, and *L. rhinocerotis*, as well as their mechanisms of action. The underlying events are, however, not fully understood, and further research is required. Overall, *H. erinaceus* appeared as the most promising for potential clinical applications, given the stronger evidence-based data provided in the reviewed studies and its relatively safer components compared to *A. muscuria* and other mushroom species. Notwithstanding, clinical models are necessary to harness the potential of mushroom species and the development of future therapeutics and preventive measures for PNI.

While only a few studies on mushrooms fit into the inclusion criteria utilised in this review, the results are not surprising, considering research on the use of mushrooms for PNI treatment has only gained wider interest in the last decade. The use of mushroom-derived treatments for peripheral nerve repair and regeneration is still underreported, and their potential source for promising drug candidates remains untapped. Several prior studies have shown that various mushrooms, such as *Hirsutella sinensis*, *Ganoderma lucidum*, and *Antrodia cinnamomea*, are capable of activating intracellular signalling kinases associated with the regeneration of peripheral nerves. Different species of Ganoderma were also reported to enhance neuritogenesis through the MAPK signalling pathway. Nonetheless, the roles of these mushrooms as potential candidates for treating PNI are yet to be explored. Apart from investigating untapped mushroom species, future studies may also investigate their effectiveness with well-established neuroregenerative properties to compare their ability to enhance peripheral nerve regeneration.

## Figures and Tables

**Table 1:** Group of keywords used for literature searches.

Group 1	Group 2	Group 3
Mushroom, Hericium *Amanita*, *Lignosus*, Alternative, Natural, Complementary	Peripheral nerve, Nerve injury, Nerve crush, Neuroprotective, Regeneration, Therapeutic, Treatment, Repair, In vivo, In vitro	Function, Recovery

**Table 2:** Search strings used for the literature search in each database.

Scopus
(Mushroom) OR (Hericium) OR (Amanita) OR (Lignosus) OR (Alternative) OR (Natural) OR (Complementary) AND (Peripheral nerve) AND (Nerve Injury) OR (Nerve crush) OR (Neuroprotective) OR (Regeneration) OR (Therapeutic) OR (Treatment) OR (Repair) OR (In vivo) OR (In vitro) AND (Function) OR (Recovery) Search within Article, Abstract, and Keywords. Year = 2010–2024, results limited to research articles = 961
**PubMed**
**Query #1**: TS= (Mushroom OR Hericium OR Amanita OR Lignosus OR Alternative OR Natural OR Complementary) **Query #2**: TS= (“Peripheral nerve” OR “Nerve Injury” OR “Nerve crush” OR “Neuroprotective” OR “Regeneration” OR “Therapeutic” OR “Treatment” OR “Repair” OR “In vivo” OR “In vitro”) **Query #3**: TS= (Functional OR Recovery) **Query #4** #1 AND #2 AND #3 and Article (Document Types) and English (Languages) Year = 2010–2024, results limited to research articles = 502
**Google Scholar**
(Mushroom) OR (Hericium) OR (Amanita) OR (Lignosus) OR (Alternative) OR (Natural) OR (Complementary) AND (Peripheral nerve) OR (Nerve Injury) OR (Nerve crush) OR (Neuroprotective) OR (Regeneration) OR (Therapeutic) OR (Treatment) OR (Repair) OR (In vivo) OR (In vitro) AND (Function) OR (Recovery) Year = 2010–2024, Results = 152,000, screening limited to the first 300 articles after sorting based on relevance

**Table 4:** Risk of bias assessment (*n* = 11).

Reference	D1	D2	D3	D4	D5	D6	D7	D8	D9	D10	Overall
Naik et al. [19]	Unclear	Yes	Unclear	Unclear	Unclear	Unclear	Unclear	Yes	Yes	Yes	Low
Wang et al. [9]	Unclear	Yes	Unclear	Unclear	Unclear	Unclear	Unclear	Yes	Yes	Yes	Low
Wong et al. [25]	Unclear	Yes	Unclear	Yes	Unclear	Unclear	Unclear	Yes	Yes	Yes	Moderate
Wong et al. [26]	Unclear	Yes	Unclear	Yes	Unclear	Unclear	Unclear	Yes	Yes	Yes	Moderate
Lin et al. [21]	Unclear	Yes	Yes	Unclear	Unclear	Unclear	Unclear	Yes	Yes	Yes	Moderate
Üstün & Ayhan [24]	Unclear	Yes	Unclear	Unclear	Unclear	Unclear	Unclear	Unclear	Yes	Yes	Low
Martínez-Mármol et al. [13]	Unclear	Yes	Unclear	Unclear	Unclear	Unclear	Yes	Unclear	Yes	Yes	Low
Farha et al. [27]	Unclear	Yes	Unclear	Unclear	Unclear	Unclear	Unclear	Yes	Yes	Yes	Low
Lee-Fang Eik et al. [23]	Unclear	Yes	Unclear	Unclear	Unclear	Unclear	Unclear	Yes	Yes	Yes	Low
Seow et al. [14]	Unclear	Yes	Unclear	Unclear	Unclear	Unclear	Unclear	Unclear	Yes	Yes	Low
Chen et al. [22]	Unclear	Yes	Unclear	Unclear	Unclear	Unclear	Unclear	Unclear	Yes	Yes	Low

Note: D1 = Was the allocation sequence generated adequately and applied? D2 = Were the baseline groups similar, or was adjustment performed for confounders in the analysis? D3 = Was the allocation concealed adequately? D4 = Were the animals housed randomly during the trial? D5 = Were the investigators blinded from the intervention administered during the experiment? D6 = Were the animals or treatment groups selected randomly for outcome assessment? D7 = Was the outcome assessor blinded?, D8 = Were missing data adequately addressed? D9 = Are the study findings free of selective outcome reporting? D10 = Is the study free of other issues that may cause a high risk of bias?

## Data Availability

No new data were created or analyzed in this study.

## References

[1] Navarro X. (2016). Functional evaluation of peripheral nerve regeneration and target reinnervation in animal models: A critical overview. Eur. J. Neurosci..

[2] Wojtkiewicz D.M., Saunders J., Domeshek L., Novak C.B., Kaskutas V., Mackinnon S.E. (2015). Social impact of peripheral nerve injuries. Hand.

[3] Li N.Y., Onor G.I., Lemme N.J., Gil J.A. (2020). Epidemiology of peripheral nerve injuries in sports, exercise, and recreation in the United States, 2009–2018. Phys. Sportsmed..

[4] Scholz T., Krichevsky A., Sumarto A., Jaffurs D., Wirth G., Paydar K., Evans G.R.D. (2009). Peripheral nerve injuries: An international survey of current treatments and future perspectives. J. Reconstr. Microsurg..

[5] Antoniadis G., Kretschmer T., Pedro M.T., König R.W., Heinen C.P., Richter H.P. (2014). Iatrogenic nerve injuries-prevalence, diagnosis and treatment. Dtsch. Arztebl. Int..

[6] Popa V.I., Racasan O.F., Margina A.C., Silviu Cortan S., Neagu P., Sebe I.T. (2016). Advances in peripheral nerve regeneration: Materials, methods, techniques. Modern. Med..

[7] Angius D., Wang H., Spinner R.J., Gutierrez-Cotto Y., Yaszemski M.J., Windebank A.J. (2012). Systematic review of animal models used to study nerve regeneration in tissue-engineered scaffolds. Biomaterials.

[8] Grinsell D., Keating C.P. (2014). Peripheral nerve reconstruction after injury: A review of clinical and experimental therapies. BioMed Res. Int..

[9] Wang E.W., Zhang J., Huang J.H. (2015). Repairing peripheral nerve injury using tissue engineering techniques. Neural Regen. Res..

[10] Yow Y.Y., Goh T.K., Nyiew K.Y., Lim L.W., Phang S.M., Lim S.H., Ratnayeke S., Wong K.H. (2021). Therapeutic potential of complementary and alternative medicines in peripheral nerve regeneration: A systematic review. Cells.

[11] Brunelli B., Gorson K.C. (2004). The use of complementary and alternative medicines by patients with peripheral neuropathy. J. Neurol. Sci..

[12] Yadav S.K., Ir R., Jeewon R., Doble M., Hyde K.D., Kaliappan I., Jeyaraman R., Reddi R.N., Krishnan J., Durairajan S.S.K. (2020). A mechanistic review on medicinal mushrooms-derived bioactive compounds: Potential mycotherapy candidates for alleviating neurological disorders. Planta Medica.

[13] Martínez-Mármol R., Chai Y., Conroy J.N., Khan Z., Hong S., Kim S.B., Gormal R.S., Lee D.H., Lee J.K., Coulson E.J. (2023). Hericerin derivatives activate a pan-neurotrophic pathway in central hippocampal neurons converging to ERK1/2 signalling enhancing spatial memory. J. Neurochem..

[14] Seow S.L.-S., Eik L.-F., Naidu M., David P., Wong K.-H., Sabaratnam V. (2015). *Lignosus rhinocerotis* (Cooke) Ryvarden mimics the neuritogenic activity of nerve growth factor via MEK/ERK1/2 signalling pathway in PC-12 cells. Sci. Rep..

[15] De Silva D.D., Rapior S., Sudraman E., Stadler M., Xu J., Alias S.A., Hyde K.D. (2013). Bioactive metabolites from macrofungi: Ethnopharmacology, biological activities and chemistry. Fungal Divers..

[16] Phan C.W., David P., Sabratnam V. (2017). Edible and medicinal mushrooms: Emerging brain food for the mitigation of neurodegenerative diseases. J. Med. Food.

[17] Choo S., Phillips R., White J., Nuelle J.A. (2023). Neuromodulators can promote nerve regeneration and accelerate functional recovery after peripheral nerve injury: A systematic review. J. Orthop..

[18] Campbell M., McKenzie J.E., Sowden A., Katikireddi S.V., Brennan S.E., Ellis S., Hartmann-Boyce J., Ryan R., Shepperd S., Thomas J. (2020). Synthesis without meta-analysis (Swim) in systematic reviews: Reporting guideline. BMJ.

[19] Hooijmans C.R., Rovers M.M., de Vries R.B., Leenaars M., Ritskes-Hoitinga M., Langendam M.W. (2014). SYRCLE’s risk of bias tool for animal studies. BMC Med. Res. Methodol..

[20] Naik A.K., Latham J.R., Obradovic A., Jevtovic-Todorovic V. (2012). Dorsal root ganglion application of muscimol prevents hyperalgesia and stimulates myelin protein expression after sciatic nerve injury in rats. Anesth. Analg..

[21] Lin C.Y., Chen Y.J., Hsu C.H., Lin Y.H., Chen P.T., Kuo T.H., Ho C.T., Chen H.H., Huang S.J., Chiu H.C. (2023). Erinacine S from *Hericium erinaceus* mycelium promotes neuronal regeneration by inducing neurosteroid accumulation. J. Food Drug Anal..

[22] Chen F., Wu M., Wu P., Xiao A., Ke M., Huselstein C., Cai L., Tong Z., Chen Y. (2021). Natural *Flammulina velutipes* -based nerve guidance conduit as a potential biomaterial for peripheral nerve regeneration: In vitro and in vivo studies. ACS Biomater. Sci. Eng..

[23] Lee K.F., Chen J.H., Teng C.C., Shen C.H., Hsieh M.C., Lu C.C., Lee K.C., Lee L.Y., Chen W.P., Chen C.C. (2014). Protective effects of *Hericium erinaceus* mycelium and its isolated erinacine A against ischemia-injury-induced neuronal cell death via the inhibition of iNOS/p38 MAPK and nitrotyrosine. Int. J. Mol. Sci..

[24] Üstün R., Ayhan P. (2019). Regenerative activity of Hericium erinaceus on axonal injury model using in vitro laser microdissection technique. Neurol. Res..

[25] Wong K.H., Kanagasabapathy G., Naidu M., David P., Sabaratnam V. (2016). *Hericium erinaceus* (Bull.: Fr.) Pers., a medicinal mushroom, activates peripheral nerve regeneration. Chin. J. Integr. Med..

[26] Wong K.H., Kanagasabapathy G., Bakar R., Phan C.W., Sabaratnam V. (2015). Restoration of sensory dysfunction following peripheral nerve injury by the polysaccharide from culinary and medicinal mushroom, *Hericium erinaceus* (Bull.: Fr.) Pers. through its neuroregenerative action. Food Sci. Technol..

[27] Farha M., Parkianathan L., Amir N.A.I.A., Sabaratnam V., Wong K.H. (2019). Functional recovery enhancement by tiger milk mushroom, *Lignosus rhinocerotis*, in a sciatic nerve crush injury model and morphological study of its neurotoxicity. J. Anim. Plant Sci..

[28] Zhang C.C., Cao C.Y., Kubo M., Harada K., Yan X.-T., Fukuyama Y., Gao J.-M. (2017). Chemical constituents from *Hericium erinaceus* promote neuronal survival and potentiate neurite outgrowth via the TrkA/Erk1/2 pathway. Int. J. Mol. Sci..

[29] Namikawa K., Honma M., Abe K., Takeda M., Mansur K., Obata T., Miwa A., Okado H., Kiyama H. (2002). Akt/protein kinase B prevents injury-induced motoneuron death and accelerates axonal regeneration. J. Neurosci..

[30] Raman J., Lakshmanan H., John P.A., Zhijian C., Periasamy V., David P., Naidu M., Sabaratnam V. (2015). Neurite out-growth stimulatory effects of myco-synthesised AuNPs from *Hericium erinaceus* (Bull.: Fr.) Pers. on pheochromocytoma (PC-12) cells. Int. J. Nanomed..

[31] Li X., Wang Z., Wang L., Walid E., Zhang H. (2012). In vitro antioxidant and anti-proliferation activities of polysaccharides from various extracts of different mushrooms. Int. J. Mol. Sci..

[32] Contato A.G., Conte-Junior C.A. (2025). Lion’s Mane Mushroom (*Hericium erinaceus*): A Neuroprotective Fungus with Antioxidant, Anti-Inflammatory, and Antimicrobial Potential-A Narrative Review. Nutrients..

[33] Charalampopoulos I., Remboutsika E., Margioris A.N., Gravanis A. (2008). Neurosteroids as modulators of neurogenesis and neuronal survival. Trends Endocrinol. Metabol..

[34] Djebaili M., Guo Q., Pettus E.H., Hoffman S.W., Stein D.G. (2005). The neurosteroids progesterone and allopregnanolone reduce cell death, gliosis, and functional deficits after traumatic brain injury in rats. J. Neurotrauma.

[35] Lu C.C., Hsu Y.J., Chang C.J., Lin C.S., Martel J., Ojcius D.M., Ko Y.F., Lai H.C., Young J.D. (2016). Immunomodulatory properties of medicinal mushrooms: Differential effects of water and ethanol extracts on NK cell-mediated cytotoxicity. Innate Immunity.

[36] Pilipenko V., Pupure J., Rumaks J., Beitnere U., Dzirkale Z., Skumbins R., Klusa V. (2015). GABAA agonist muscimol ameliorates learning/memory deficits in streptozocin-induced Alzheimer’s disease non-transgenic rat model. SpringerPlus.

[37] Voynova M., Shkondrov A., Kondeva-Burdina M., Krasteva I. (2020). Toxicological and pharmacological profile of *Amanita muscaria* (L.) Lam.—A new rising opportunity for biomedicine. Pharmacia.

[38] Savickaitė E., Laubner-Sakalauskienė G. (2025). Emerging Risks of Amanita Muscaria: Case Reports on Increasing Consumption and Health Risks. Acta Medica Litu..

[39] Meisel E.M., Morgan B., Schwartz M., Kazzi Z., Cetin H., Sahin A. (2022). Two Cases of Severe *Amanita Muscaria* Poisoning Including a Fatality. Wilderness Environ. Med..

[40] Caillaud M., Chantemargue B., Richard L., Vignaud L., Favreau F., Faye P.-A., Vignoles P., Sturtz F., Trouillas P., Vallat J.-M. (2018). Local low-dose curcumin treatment improves functional recovery and remyelination in a rat model of sciatic nerve crush through inhibition of oxidative stress. Neuropharmacology.

